# Phosphorus-carrying cascade molecules: inner architecture to biomedical applications

**DOI:** 10.55730/1300-0527.3570

**Published:** 2023-06-23

**Authors:** Anbazhagan THIRUMALAI, Noureddine ELBOUGHDIRI, Karthick HARINI, Koyeli GIRIGOSWAMI, Agnishwar GIRIGOSWAMI

**Affiliations:** 1 Department of Medical Bionanotechnology, Faculty of Allied Health Sciences, Chettinad Hospital and Research Institute (CHRI), Chettinad Academy of Research and Education (CARE), Chennai, TN, India; 2 Department of Chemical Engineering, College of Engineering, University of Hail, Hail, Saudi Arabia; 3 Department of Chemical Engineering Process, National School of Engineers Gabes, University of Gabes, Gabes, Tunisia

**Keywords:** Dendrimers, phosphorus dendrimers, biomedical applications, theranostic cascade, drug delivery

## Abstract

Cascade molecules are nearly uniform-sized macromolecules of small molecules or linear polymer cores built around symmetric branching units. A wide range of biological properties can be achieved with phosphorus-containing dendrimers, depending on their terminal functions, ranging from biomaterials to imaging, drug delivery, and acting as a drug by themselves. This feature article presents significant examples of phosphorus-containing dendrimers used to develop biochips, support cell cultures, carry or deliver biomacromolecules and drugs, bioimaging, and combinational benefits. Because of the thermal stability, ferrocene function, and physical and chemical properties of phosphorus, dendrimers show greater rigidity, mobility, and strength. These dendrimers will be discussed as having a favorable effect on cell growths, especially on neuronal cells, as well as human immune cells like natural killer cells and monocytes, which have a crucial part in preventing cancerous and viral infections. Several phosphorus dendrimers are effective as drugs by themselves (drug per se) and show their activity against neurodegenerative diseases, cancer, inflammation, ocular hypertension, and transmissible spongiform encephalopathies (TSEs) in both in vivo and in vitro. The present review discusses the synthetic route, fabrications, and biomedical applications of phosphorus-containing dendrimers. The toxicity of these dendrimers was also reported.

## 1. Introduction

The recent progress in nanomedicine brought a concept of using a single platform that combines diagnosis and therapeutic called theranostic particles [1,2]. Over the past few years, theranostic nanomaterials that can simultaneously track and cure diseases have been extensively studied because monitoring, biocompatibility, biodistribution, and therapeutic potentials of the administered therapeutic agent, as well as detecting the target tissue, have been addressed by the theranostic particles [3–5]. Several polymeric nanodevices have been developed for the delivery of drugs and genes against cancer, besides for imaging and tracking tumor cells through specific biomarkers [6]. There are numerous linear polymers that have been accepted in various clinical applications, including polyethylene glycol, polycaprolactone, and poly (lactic-co-glycolic acid) [7,8]. However, the nanomedicines have some shortcomings, such as polydispersity, nonspecific binding, poor biodistribution, and low loading capacity [9,10]. Additionally, the reticuloendothelial system recognizes and engulfs liposomes and micelles, resulting in their low therapeutic index. Dendrimer offers a promising platform to address all these issues. There are dendrons within dendrimers, which are arms that stem from the core. Dendrimers are generally made up of three major components, namely the central core, repeated units attached to the central core, which are typically homocentric layers known as generations, and the terminal functional groups, which determine the pharmacokinetic profiles and the biocompatibility of the dendrimer [11]. The chemical structure of dendrimers as well as their physical characteristics can be classified into several subcategories based on their chemical composition.

The bottom-up approach, which is based on the development of tools from the molecular level up, is now considered to be the most promising path for the future of technology to serve biomedical applications. A considerable amount of attention is being paid to small molecules in this context, and special attention is being given to a particular class of polymers known as dendrimers. Dendrimers, otherwise called cascade molecules, are highly branched three-dimensional macromolecules where their hyperbranched structures are synthesized step-by-step, consisting of repetitive monomeric units to ensure reproducible structure. The study of dendritic macromolecules that originate from a central core and consist of well-defined branching units constitutes an important area of research [12]. Dendrimers are considered materials in their solid form, or they can be used to create new materials or to modify the surfaces of standard materials. In addition to purely organic dendrimers, dendrimers containing phosphorus atoms at every branching point occupy a special position. Aside from their simplicity, they are also distinctive by their rich chemistry, which is often characterized by highly reactive end groups, such as aldehydes or phosphorus-chloride (P (S)Cl_2_).

Depending on the surface terminal function, they vary in the types and synthetic processes used for dendrimers, while new properties can be obtained by modifying the terminal functions uniformly. Some dendrimers have different types of terminal functions, and each function provides its own property. There are two types of surfaces, namely, bifunctionalized dendrimers: stochastic functionalization and precise bifunctionalization [13]. Organic dendrimers are composed of branching units with amines, like poly (propylene imine) (PPI) and poly (amidoamine) (PAMAM) dendrimers, whereas inorganic dendrimers contain inorganic atoms like phosphorus and silicones at their branching units. Phosphorus dendrimers contain either P (S)Cl_2_ (phosphorus-chloride) or aldehydes in their terminal groups. This review discusses phosphorus-containing dendrimers and their roles in nanomedicine in detail. The surface modification of phosphorus dendrimers improves the biocompatibility of the material, and various toxicity assays on different cells supports its cytotoxicity. Then in vivo and in vitro imaging using fluorescent dendrimers and charges of dendrimer for the biological entities or drug delivery were also reported. Finally, various examples of phosphorus dendrimers are used as a drug by themselves (drugs per se).

## 2. Synthetic strategies

Method for the preparation of dendrimers has been reported by several researchers. In 1978, Vogtle et al. first synthesized the PPI [poly (propylene imine)] dendrimers by an iterative method. The divergent approach was applied in synthesizing PAMAM dendrimers and arborols (type of dendrimer structure) in 1985. Later, Hawker and Fréchet reported a convergent approach to synthesizing dendrimers.

### 2.1. Convergent method

**Figure 1:**
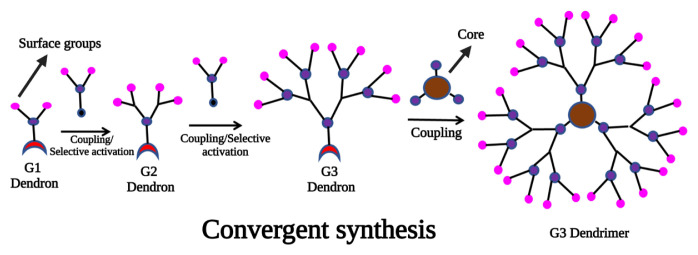
**A** schematic approach to the synthesis of dendrimers based on convergent synthesis.

Convergent growth is initiated by joining the end groups of the monomer to the ends of the initial monomer, and by doing so, the monomer grows outward. As a result of the complete coupling, the dendritic fragment, usually shaped like a wedge, can be activated by a single functional group located at its focal point, called a dendron [14]. The dendrons formed in this way can be attached to a polyfunctional core through their focal point to create globular multidendron-based dendrimers once enough repetitions of this process are carried out. First generation dendrimers (G1) were generated from a core with functional groups available for reactivity with monomers (Figure 1). In the next step, the inactive groups of monomers are detached, allowing them to attach to further monomers, resulting in the formation of dendrimers. As the generation number increases, the monomer’s contribution to the mass of the product decreases exponentially, despite the molecular weight of the dendron effectively doubling at every coupling step. With each additional generation, the mass of the sample decreases due to nonquantitative coupling yields and purification losses [14]. Due to the fact that the coupling reaction occurs at the focal point of the growing dendron, steric inhibition is usually a problem when preparing quite large dendrimers (typically those over the sixth generation), resulting in decreases in yields. With convergent synthesis, only a few transformations are required per molecule during activation and coupling steps. Thus, a relatively small amount of reagent is required for the synthesis of high-generation dendrimers, as opposed to the massive amount required for divergent synthesis [15,16].

There are a number of parameters that need to be met for convergent synthesis to be successful, including the use of monomers that can be activated and coupled in a high yield, and whose products can be readily separated from excess starting material and byproducts [14]. As a result of the relatively low number of coupling reactions required at every growth step, convergent synthesis provides greater structural control than divergent synthesis, resulting in dendritic products that are both pure and functionally versatile. Poly (aryl ether), developed by Fréchet et al., and poly (aryl alkyne), developed by Moore and his team, are the most widely used convergent syntheses [17]. One of the most attractive features of the convergent synthesis is its ability to create well-defined unsymmetrical dendrimers by precisely placing functional groups throughout the structure, by modifying the focal point or the chain ends selectively, and by preparing well-defined focal points [18,19]. Its commercialization, however, is currently limited, mostly due to the difficulty in scaling this up compared to divergent synthesis.

### 2.2. Divergent method

**Figure 2:**
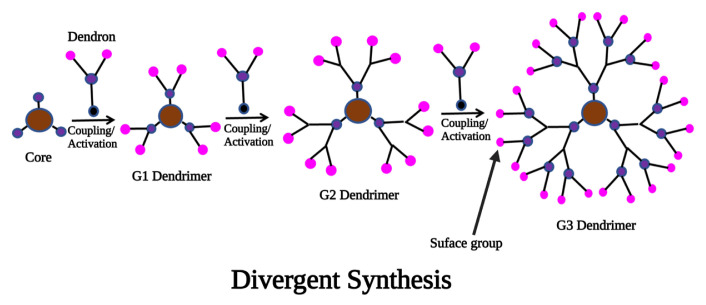
Schematic illustration of divergent strategies for the synthesis of dendrimers.

In this method, the synthesis of dendrimers starts from the monomers (outside) to the core (inside). Each coupling site introduces a new lurking branch point, which results in an increase in peripheral functionalities by the reaction of the peripheral functionalities with the monomer’s complementary reactive group. Polymerization of uncontrolled hyperbranched polymers is prevented through the peripheral functionalities of each monomer. It is possible to activate the latent functionalities after the first coupling reaction has been completed to produce a new layer of peripheral functional groups that can be coupled to additional monomers [20]. Peripheral groups can be activated by converting them into reactive groups, coupling with other molecules, or removing protecting groups. The number of reactions at the periphery exponentially increases with the repetition of the coupling and activation steps; therefore, an abundance of reactants is required for both reactions to occur. Simple distillation, precipitation, or ultrafiltration may be able to separate the macromolecular dendrimer structure from the reaction mixture with an excess of reactants due to the difference in molecular weight. The advantage of divergent synthesis is with the lower purity, dendrimers will get a higher yield, or we can say that to get a higher yield, they were compromised with their purity (Figure 2). The lesser purity of the synthesis is achieved by any one of the intermolecular and intramolecular cyclizations, missing repeated units, ester hydrolysis, and retro Michael reaction. Because of this approach, the syntheses of dendrimers are used worldwide on a commercial scale and are most useful.

Using the divergent approach, the quantity of dendrimer sample essentially doubles with every generation increment if coupling and activation steps, reagents, and reaction conditions are appropriate [14]. However, since the number of coupling reactions is increasing exponentially with each generation, there will also be an exponential increase in the possibility of incomplete functionalizations or side reactions occurring with each generation. There are many things that can be done to remove the monomer, such as cyclizing the monomer or incomplete reaction, but it may not be able to remove any flawed molecules because their structure is identical to the product to be isolated or wished to separate [21]. To prevent the growth of smaller dendritic impurities, meticulous measures must be taken to ensure that the activating agent itself cannot initiate new growth. Poly (amidoamines) (PAMAM) and poly (propylene imine) dendrimers seem particularly noteworthy among the many divergent syntheses studied to date [22,23].

## 3. Types of dendrimers

### 3.1. Carbosilane dendrimers

Synthesis and characterization of tetrahedral Si core are grown as carbosilane dendritic macromolecules. As a result of silicon chemistry, dendrimers can be synthesized by nucleophilic molecules quickly accessing electrophilic silicon (Si+). Functionalizing their surface with ionic groups converts them into water-soluble compounds with a hydrophobic inner skeleton. It is possible to introduce numerous intriguing and useful organic, inorganic, and organometallic substituents through the use of reactive peripheral groups such as Si-H, Si-Cl, Si-CH_2_CH=CH_2_, and Si-CH=CH_2_ [24].

Furthermore, carbosilane dendrons with biomedical applications have been synthesized and described, including antibodies against influenza viruses. A second and third generation carbosilane dendron was reported to be effective in functionalizing the external surface of mesoporous silica nanoparticles [25]. In in vitro studies, nonviral oligonucleotides are carried by hybrid materials to deliver into human osteoblast cell lines. A dendritic amphiphile derived from peripheral PEG and carbosilane dendrimers has been synthesized and tested as a drug delivery system. Carbosilane dendrimers with small sizes have efficiency for carrying the small RNAs and are used for the treatment of HIV [26].

### 3.2. Polypropylene imine dendrimers or PPI dendrimers

A modified Vogtle method was used by Wörner/Mülhaupt and de Brabander-Van den Berg/Meijer 15 years later to synthesize polypropylene imine dendrimers (PPI). Several dendrimer generations with well-defined structures and morphologies were synthesized by Tomalia, Fréchet, and Newkome shortly after that [27]. The exciting breakthrough in the synthetic engineering of dendrimers led to the evolution of different synthetic routes for the synthesis of different generations of dendrimers. For example, PPI dendrimers were synthesized by the divergent method. Sodium borohydride (NaBH_4_) in the presence of cobalt (II) acetate was used to reduce acrylonitrile when added to primary mono- or oligodiamines. Generation 1 poly (propylene imine) dendrimers were synthesized by adding acrylonitrile and 1, 4-diaminobutane mixture at 0 °C for 30 min. Rotary vacuum was another method used to remove the excess acrylonitrile, and dendrimers were obtained as the oily viscous light solution [28].

Electrostatic forces can attach several negatively charged drug molecules to PPI dendrimers with cationic groups on the surface. They form complexes with drugs, nucleic acid, and peptides that are widely used in vitro and in vivo for carrying biomolecules [26]. Positively charged dendrimers pose a problem due to their high toxicity associated with the generation of dendrimers and a growing number of cationic surface groups (amino) that can damage cells [29]. The surface group modification of the PPI dendrimers, especially with sugar, will help to reduce toxicity. PPI dendrimers modified with maltose have good biocompatibility, and their neutral and cationic surfaces can interact with cationic and anionic molecules, respectively [30].

### 3.3. Phosphorus dendrimers

Phosphorus dendrimers belong to nanodevices family, and they were developed mainly for active delivery, contrast-bearing agent for imaging, and drug delivery. In the field of therapeutic area, phosphorus dendrimers have many in vivo applications; as a drug for noninvasive in vivo imaging, anticancer agents, antiinflammatory agents, and treating ocular hypertension, neurodegenerative diseases, and infections [31].

### 3.4. PAMAM dendrimers or poly (amidoamine) dendrimers

In biomedical applications, PAMAM (poly (amidoamine)) dendrimers have peptide bonds inside branches, and were the first synthesized dendrimers, that were primarily used in clinical studies. PAMAM dendrimers have a high surface area at the outer end functional groups (surplus) and are hyperbranched and monodisperse polymers [32]. The immobilization of molecules or biorecognition species occurs by the amine and amide functional groups. Among dendrimers, PAMAM is arguably the most prominent and characterized, and it is also the first commercialized dendrimer [33].

Under an inert atmosphere, 25 mL of methanol was treated with a mixture of methyl acrylate and diethylenetriamine for five days [34]. After that, evaporation of the volatile compounds was done and G1-dendrimers were obtained with 5-OCH_3_ terminal group PAMAM dendrimers. Biosensors, drug delivery, catalysis, and sensors are just some of the application areas in which PAMAM dendrimer has played a major important role.

### 3.5. Radially layered poly (amidoamine organosilicon) (PAMAMOS) dendrimers

In 1990, an odd new class of dendrimers called PAMAMOS was discovered by Dr. Petar Dvornic and his team at Michigan Molecular Institute [35]. This dendrimer was an inverted monomolecular micelle containing a nucleophilic poly amidoamine and hydrophilic organosilicon component. Poly (amidoamine organosilicon) dendrimers could form different complexes and were capable of binding a variety of molecules, making them useful in various application fields, including chemical catalysis, electronics, nanolithography, and photonics [36].

### 3.6. Chiral dendrimers

In macromolecular systems, chiral dendrimers play an essential role in understanding handedness. In chiral dendrimer: chiral molecule interactions, kinetics, and optical measurements are used, while enantioselective synthesis and chiral recognition are used for smaller molecules [37].

## 4. Chemistry of phosphorus dendrimers

**Figure 3:**
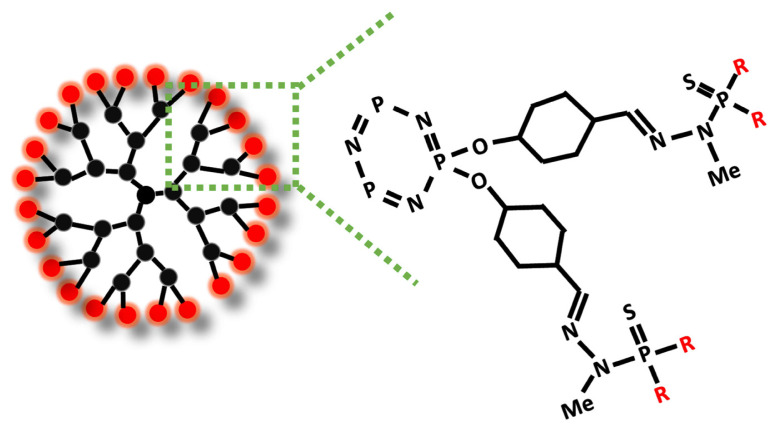
Synthesis of phosphorus dendrimer.

The chemical construction of organized matter plays a key role in dendrimers. Cohydrolysis and polycondensation of dendrons containing hydrolyzable Si (OEt)_3_ groups at the core and sufficient Si (OEt)_4_ and water equivalents. Phosphorus-containing dendrimers create a multitype network by supramolecular assemblies, dependent on the types of end groups, as shown in Figure 3 [38]. Thin-layer dendrimers induce the formation and growth of cluster crystals and protect them against decomposition. In materials science, thermal stability is a very important characteristic of any substance used for applications. Dendrons and dendrimers with numerous end groups, cores, and generations were analyzed, and it showed no influence on the thermal stability between generations [39]. The surface of the dendrimers modified with existing materials can be an ionic anchorage (reversible) or covalent anchorage (irreversible). The addition of several monolayers of dendrons to a surface, eventually together with other components, can be accomplished through covalent or ionic interactions when the dendrimers are charged on its surface used [40].

### 4.1. Properties of phosphorus dendrimers

Thermal stability is one of the preeminent properties to study when using a compound for application. In aldehyde end-groups, dendrimers thermogravimetries were analyzed for 1–6 generations. It was found that pyridinium end-groups have an important influence on the stability of pyridinium end-groups at 225 °C and 376 °C for ones with ferrocene end-groups. This showed the internal structure of the dendrimer was stable [41]. The physical properties of dendrimers containing aldehyde end-groups were investigated, and the standard differential scanning calorimetry as temperature-modulated calorimetry was used, and it showed the metastability of the dendrimers. There was a decrease in molecular mobility in the solid phase from 1–3 generations, and 3–5 higher generations were characterized by greater rigidity due to the molecular mobility in the solid phase [42]. An experiment to study the effects of ‘burying’ particular functions inside dendrimers showed the same behavior. In the study of ferrocene functions within dendrimers, electrochemical techniques were employed to study dendrimers that had ferrocene functions on the surface because they were gradually buried. Compared with the inside dendrimer, ferrocene-linked inside the dendrimer showed a higher rigidity due to less electrolysis ability [43].

### 4.2. Fabrication methods of phosphorus dendrimers

Using phosphonium at each branch point was the first phosphorus dendrimers reported by Engel et al. in 1990, but these were never found to be useful biologically. By repeating chain elongation and branching steps, various dendrimers with adenosine and thymidine building blocks were synthesized [44]. The water-soluble phosphorus dendrimers were synthesized mainly because of the phosphonium branching points in their internal structure. The 2nd generation dendrimers were synthesized in the solid phase by either a convergent or divergent process based on thymidine and bridged by phosphoramidite was also soluble mainly because of their internal structure. Up to 3rd generation, the synthesis of dendrimers was carried out, but the 1st and 2nd generations were soluble in water [45].

Researchers have developed the improved methods to simplify the synthesis of dendrimers, as the synthesis was brief [46]. Initially, the multigeneration synthesis occurred in one step, but recently scientists have presented a new method for rapidly multiplying the number of functional groups by utilizing CD5 and AB5 monomers. This method of dendrimers had two varieties of repetitive units, like layered dendrimers [46]. Polycondensation of a one-step method of using AB_2_ type of monomers was used to synthesize hyperbranched polymer. This class of polymers can be considered as a potential alternative to dendrimers due to the relatively high degree of branching (0.83–0.85), even though the polydispersity of these polymers was higher (1.16–2.51) compared to dendrimers from the same repeat unit (1.008 for 3rd generation) but their degree of branching could not be said to be overall high (0.83–0.85). The phosphorus dendrimers were also functionalized with biocompatible functions by this method, after which they were adapted for biological applications and were described in 1994 for the synthesis of large phosphorus-containing dendrimers [47]. The substitutional reaction of Cl linked by 4-hydroxybenzaldehyde in phosphorus was the first synthesis process, and then H_2_NNMeP (S)Cl_2_ was condensed with aldehydes. These quantitative steps created a new generation by ensuring the multiplication of the terminal groups and generating H_2_O and NaCl as byproducts. P (S)Cl_3_ was used as the 1st-G core, followed by the 7th-G, 8th-G, and finally, the 12th generation 3a-G12 [48].

## 5. Applications of phosphorus dendrimers

**Figure 4:**
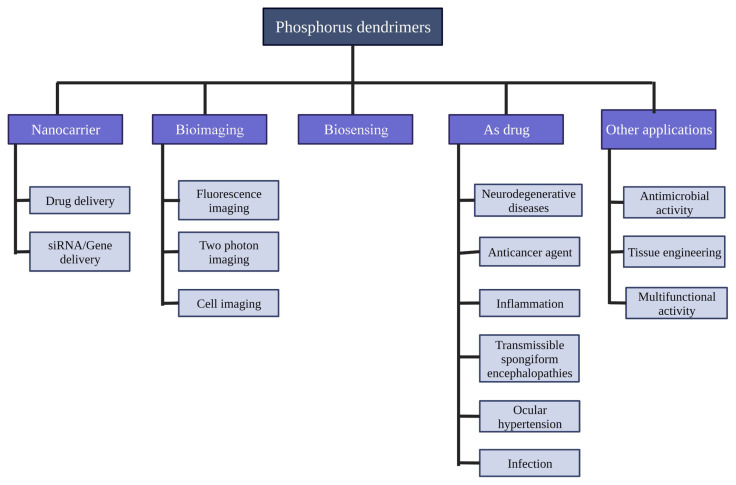
Biomedical applicants of phosphorus dendrimers.

Among the application of various fields, researchers have developed materials for biology and many chemistry-related fields (Figure 4). The large size of the dendritic catalysts could make it an interesting candidate for recovering and reusing, especially if ligands or metals of higher cost were required. There was no difference in catalytic efficiency between dendrimeric and monomeric catalysts, but sometimes the 3G-dendrimeric catalysts can be reused more than twice without significantly reducing their catalytic efficiency. Dendrimer properties were strongly influenced by their size, as shown in various examples in materials science. The thiol end-group dendrimer’s size and density were highly dependent on the crystal size and shape [49]. The dendrimer size was also the most important criterion in different parts of biology, like transfection experiments. Tertiary ammonium end group dendrimers with 1–5 generations were tested in mammalian cells for transfecting the luciferase plasmid. Generation 1–3 increased their efficiency dramatically, and generating 3–5 reached a plateau. Furthermore, it had been emphasized that the fourth generation of phosphorus dendrimers yielded the best antiprion results, highlighting the generation’s importance on the properties of the compound [50].

### 5.1. Payload delivery

**Figure 5:**
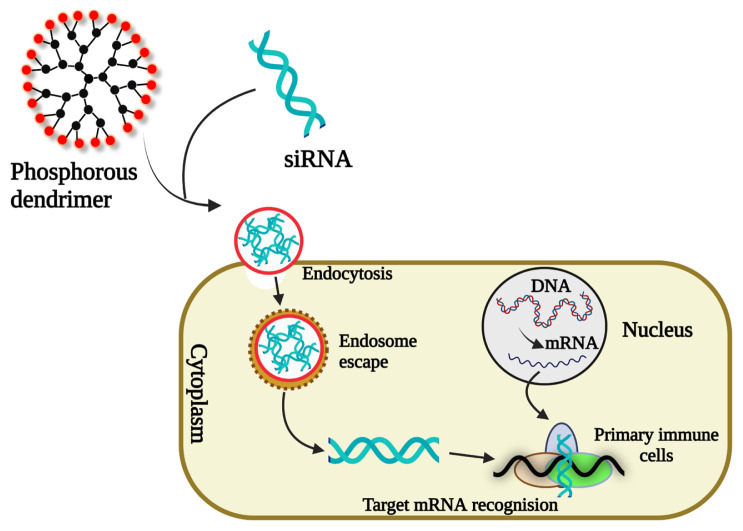
Phosphorus dendrimer for siRNA delivery into primary immune cells (T cell, NK cell, and monocytes/macrophages).

Spataro and his team made the first experiment—in vivo ocular delivery of cationic phosphorus dendrimers for rabbits. New phosphorus-containing dendrimers have been synthesized from generation 0 to generation 2 using a carboxylic acid terminal group and quaternary ammonium salt as a core. Using this dendrimer, the neutral form of carteolol (to treat glaucoma antihypertensive) was encapsulated to form ion pair species for successful delivery with improved solubility [51]. In 2003, Le Berre and his team made an attempt to separate DNA in microarray with the use of phosphorus dendrimers. This microarray technique was highly dependent and also known as safe and successful, because of many factors like convenience of cDNA targets fixed on the surface of DNA probe and surface chemistry parameters. An amino-modified DNA probe was covalently bound to amino-modified dendrimers attached to amino-silanized glass slides by covalent attachment [52]. For example, the company Dendris is currently developing a multiplex method for detecting several bacteria in real-time; food safety and sexual diseases, that were commonly implicated by ten pathogens [53]. In PAMAM and Phosphorus dendrimers, anionic oligomers increased the capacity of DNA delivery into cells by adding into dendrimers they were mixed with plasmid DNA (Figure 5). Depending on the size, structure, and charge of oligomers, the DNA/dendrimer penetration efficacy would increase [54].

The use of dendrimers in drug delivery has been a topic of interest for more than two decades. In dendrimers, the delivery and transport of drugs are carried out by electrostatic interactions, covalent bonds, and entrapment inside the dendrimers. Fipronil grafted phosphorus dendrimer formed by labile imine function, and its release in the air was 12% (1st generation) and 37% (3rd generation), respectively, after 35 days [55]. Silica nanoparticles grafted into the dendrimers and PEG terminal groups were functionalized on their surface. They displayed slow release of Ag nanoparticles and antibacterial activity [56]. Solassol et al. have demonstrated that cationic phosphorus dendrimer (pd) G4 has antiprion activity on the cells that were infected with the scrapie from prions. Scrapie brain homogenate was i.p. injected (intraperitoneally) into the infected mouse. Every two days, the mouse was i.p. injected (postinoculation) starting from day 2 to continuously for day 30 with 50 mg or 100 mg of pd-G4 dendrimers per mouse for two different groups, respectively. After 30 days of injection, the mice were sacrificed, and analysis of the spleen of mice revealed that 66% or 88% of the PrP^sc^ accumulation was inhibited by the pd-G4 dendrimers. These molecules were highly bioavailable and showed relevant potential for prion therapeutics for postexposure prophylaxis [57].

The interaction with a similar principle was recently used for carteolol delivery; there was no in vivo assay for the ion pair species 7-Gn and 8-Gn for delivering antihypertensive drugs into rabbit eyes for glaucoma treatment. Because the corneal epithelium of the eyes was quasiimpermeable, to deliver the drug into the inner eye structure, long residence times were necessary to increase the drug’s bioavailability. To enhance the bioadhesion, water-soluble polymers were added to the solution, which would increase viscosity; this was the common method for bioavailability improvement, but this may induce a vision disturbed temporarily [58]. Our specially engineered dendrimers were intended to fulfill two criteria: limitation in the formulation of chemical entities and interaction with carteolol. New dendritic compounds were synthesized; it had a carboxylic acid group at the terminal end and an ammonium salt at its core to interact with amino function of carteolol. The higher generations, like 11-G1 and 11-G2, were not fairly soluble in water, but 11-G0 was an ion pair species fully soluble in water. 11-G0 to 11-G2 compounds were installed in rabbit eyes, and no annoyance was noted even after several hours. When the quantities of carteolol entrapped in generation zero were measured in the clear fluid of eyes, a carteolol entrapped in 0 generations was practically identical to carteolol alone. When compared with carteolol alone, the amount of carteolol absorbed inside the eyes was 2.5 times greater than expected, because the 2nd generation solubility was very low. Despite the solubility issue, these observations indicated that this type of drug delivery could be biocompatible and useful [51]. Dendriplexes associated with TNF-a siRNA were synthesized with cationic amphiphilic phosphorus dendrons developed by Yu et al. In order to form the dendrons, ten pyrrolidinium end groups were bonded together with an aliphatic chain of C17. As a result of the dendriplexes, siRNA was protected from nuclease-driven degradation, and macrophage uptake was promoted [59]. To achieve successful mRNA delivery while maintaining a low toxicity profile, Joubert et al. modified PAMAM dendrimers with lysine residues as a site-selective anchor. Cy5-EGFP mRNA was then loaded onto modified dendrimers using bulk mixing controlling different nitrogen-to-phosphate ratios to facilitate endosomal escape and improvement of cellular delivery [60].

### 5.2. In vitro drug delivery using phosphorus dendrimers

Dendrimers 2-Gn (n = 1, 2) were covalently grafted with acyclovir for herpes treatment, and there were no details about its use as a drug delivery vehicle for treating herpes reported earlier [61]. In order to deliver drugs, covalent grafts must strike a delicate balance between the ability to break the delivery link and the stability to reach the target. As we showed, the pesticides were grafted to 3a-Gn dendrimers (0–4th generation); this second aspect could be difficult to induce the covalent interactions [62]. It was also possible to use ionic interactions with different effects, like lipophilic interactions, as an alternative to ionic interactions.

The 4a-Gn and 4-Gn types of carboxylic acid-ended dendrimers have amphiphilic galactosylceramide (galβ1cer) analogs that responded to aminolactitol. Gp120 viral envelope proteins were present on the surface of the HIV-1 cells, and galβ1cer binds to the V3 loop region of proteins [55]. In order to prevent the infection of cells, a chimera of galβ1cer was synthesized that interacted mightily with gp120; thereby, galβ1cer activity was inhibited. At first, aminolactitol was mixed with carboxylic-ended dendrimers obtained by the ion pair assemblies.

The previous method was applied to build dendrimers with hexafunctional core, and carboxylic acids ends were from 4-G1, resulting in 7-G1, while those with phosphonate end afforded in particular 8-G1 [63]. The dendrimers clearly identified the core functionality impact for the series 7-Ga and 7a-Ga. The bioactivities did not depend on the generations; it depended on the core. According to inhibitory assays, the alkyl chain length affected the efficiency of the inhibitor against phosphonate dendrimers of type 8-G1 [64].

### 5.3. Positively charged dendrimers for delivery

Synthesis of phosphorhydrazone dendrimers was executed by two repeating analytical steps: 4-hydroxybenzaldehyde was substituted with P–Cl functions, and condensation of aldehydes with H_2_NNMeP (S)Cl_2_ phosphorhydrazone. A hexafunctional cyclotriphosphazene core has been used as a starting point for the eighth generation of reactions, and a trifunctional P (S)Cl_3_ core has been used as a starting point for the twelfth generation of reactions. Triethylammonium-terminated positively charged phosphorus dendrimers were useful not only in biology but also in materials chemistry as nanotubes or microcapsules, and for functionalizing silica [12].

Positively charged PAMAM dendrimers were used early to deliver nucleic acid or DNA and to carry plasmid into the mammalian cells; positively charged phosphorus dendrimers were used first. The generation of dendrimers increases shows that efficiency also increases; they were used to deliver the fluorescent probe 8-anilino-1-naphthalene-sulfonate, p-GFP (plasmid of green fluorescent protein), and anticancer drug cisplatin successfully [65]. The second generation fluorescent analog were used for the transfection experiment of p-GEF and fluorescent oligonucleotide and used for the DNA interaction study [66]. The dendrimers delivered the antisense phosphorothioate oligodeoxynucleotides, gene silencing, and siRNA into PBMC and inhibited HIV-1 replication effectively. The HIV-based peptides were delivered for the development of the HIV-1 vaccine, and genes were delivered against the HIV-1 by the transfection experiment [67]. Schwann cells with doxycycline-regulated GDNF expressions provided by vectors, that were carried by the fourth generation PAMAM dendrimers, and phosphorus dendrimers with the NH_3_^+^ and NHEt_2_^+^ terminal groups. These were injected into the injured peripheral nerves of rats [68].

### 5.4. Negatively charged dendrimers for delivery

Negatively charged dendrimers are capable of interacting with the surface by noncovalent interactions [69]. N-hexadecylamino-1-deoxylactiol with carboxylic acid functions interacted with phosphorus dendrimers, affording both cationic and anionic (catanionic) dendrimers having galactosylceramide (cellular receptor involved in HIV infection at early stages) [70]. Instead of carboxylic acid derivates, phosphonic acids attached at the end of dendrimers were accomplished in analogous experiments [64]. These catanionic dendrimers were characterized physicochemically in order to determine the limitation of biological properties that influenced self-association or correlation. Phosphorus dendrimers deliver the antihypertensive drug carteolol against glaucoma using the same concept of delivery. No irritation was observed when the resultant association in water was dropped into the eyes [51].

### 5.5. Bioimaging

Fluorescence is a universal tool used for regular practice in life science like subcellular, molecular, and cellular imaging for a long period. Mostly water-soluble dendrimers and fluorescent probes were used for these imaging properties because, in dendrimers, fluorescence can be placed in different parts like in some terminal functions, core, contradictory with desired unicity, and any one layer of branches [71]. All possibilities of phosphorus dendrimers for fluorescence and bioimaging have been tested.

In human epithelioid and cervical carcinoma cell lines, 5th generation phosphorus dendrimers with octafunctional phthalocyanine core and ended with ammonium groups were able to enter inside, and it was detected by fluorescence [66]. The circular dichroism analysis of a maleimide fluorophore correlated with BACE-GFP (plasmid DNA) has shown to disrupt DNA with helical B-type structure, and has been associated with ammonium groups second generation dendrimers [72]. In addition to julolidine derivatives, azabiscarboxylate, azabisphosphonate, or vinylcarboxylate terminal groups were used to functionalize first generation dendrimer [73]. Scientists monitored the preformation of a dendrimer with azabisphosphonates or without azabisphosphonates (with vinylcarboxylates, and azabiscarboxylates), synthesized by fluorescent compound and observed their penetration into the human monocytes [74]. In order to monitor the monocytes, a fluorescein derivative was stochastically functionalized by azabisphosphonate groups in dendrimers [75].

Fluorescence from two-photon excited fluorophores was excited by the simultaneous absorption of two photons instead of one (as is the case with standard excitation) [76]. Focused penetration and deeper penetrations were the positive outcomes for imaging. The 2P-fluorophores were synthesized as dendrimers from the core (2-triphosphazene rings) and terminated by ammonium groups and have been used for imaging a rat olfactory bulb’s vascular network and a tadpole’s blood vessels [77]. All of the TPA (two-photon absorbing) fluorophores on the surface of the dendrimers were associated via phenols, which was the easiest way to modify the P (S)Cl_2_ terminal functions. A blue-emitting quadrupolar fluorophore based on a fluorene core was used in the very first example of TPA fluorophores that were functionalized on phosphorhydrazone dendrimers, and it was functionalized by phenol. The technique was highly attractive for biological imaging, especially of living animals, because it had spatially confined excitation and intrinsic three-dimensional resolution and an increased penetration depth in tissues with less photodamage due to near-infrared (NIR) excitation. Quantum dots have been shown to provide an effective alternative to fluorescent labels, but these systems have several disadvantages, such as blinking and toxicity. In order to fabricate organic nanodots, a large number of TPA chromophores were grafted onto the periphery of phosphorus dendrimers.

### 5.6. Fluoroprobes

The application of phosphorus dendrimers to improve the erection of fluorescent analogs for monitoring drug and gene delivery have been explored [78]. Maleimide-type fluorophore used water-soluble phosphorus dendrimers linked to its core, and its cytotoxicity was tested in A549 and HeLa cells. It showed low toxicity after 48 h than after 24 h [79]. An analysis of circular dichroism (CD) with plasmid DNA (BACE-GFP) indicated that this dendrimer might disturb B-type structures of helical DNA. Electrophoresis measured the firmness of the dendriplex with BACE-DEF. Fluorescent phosphorus dendrimers had a stable fluorescence, making it possible to trace transfected cells and study their functions and fates. It complemented and supported the existing paradigms on blood-borne phagocyte retention and functioned in models of spinal cord injury, when dendrimer-labeled macrophages were transplanted in vivo. A macrophage’s metabolic status could be monitored in vitro using dendrimers by monitoring their emission profile, and providing a useful tool for tracking macrophage physiological status in vivo [80]

### 5.7. Drug per se

Some dendrimers may also be considered drugs on their own (drug per se), in addition to their properties as noncovalent carriers. In some cases, the properties were enhanced by the specific function when it was linked to dendrimers.

#### 5.7.1. Against neurodegenerative diseases and transmissible spongiform encephalopathies

There are several diseases caused by the infectious prion protein PrP^Sc^, including Gerstmann-Sträußler-Scheinker syndrome, and Creutzfeldt-Jakob disease (CJD), while sheep and goats suffer from scrapie, and cows suffer from bovine spongiform encephalopathy (BSE) [81–83]. Cationic phosphorus dendrimers strongly induce prion replication in mice infected with scrapie and in cell culture also. PrP^Sc^ was rapidly removed by PD-G3 (48 diethyl amine end group), PD-G4 (96 diethyl amine end group), and PD-G5 (192 diethyl amine end group) in neuroblastoma N2a cell infected with scrapie with IC50s of 45 nM, 75 nM, and 600 nM, respectively. The concentration of these phosphorus dendrimers did not affect the growth rate or morphology of the cells, indicating that they were not cytotoxic. The amount of BSE prion strains and preexisting splenic PrPSc was decreased by the 3rd and 5th generation phosphorus dendrimers binding to PrP. The 4th generation dendrimers decreased the accumulation in the spleen up to 66% and 88% at the dose of 50 μg and 100 μg, respectively in C57BL/6 mice scrapie model. Generation 4 phosphorus dendrimers were intraperitoneally administrated from day 2 to 30 days, postinoculation with two days intervals; and C57BL/6 mice were sacrificed after 30 days of infection. When the phosphorous dendrimers almost reached the infected brain, the animals’ spleen were monitored by an in vivo experiment [84].

Positively charged phosphorus dendrimers were used first as a drug per se against PrP^Sc^ (scrapie form of prions), which is responsible for transmissible spongiform encephalopathies development. A strong antiprion activity was observed by 4th generation dendrimers; it decreased multiple prion strains of PrP^Sc^, including scrapie-infected cells and mad cow disease at noncytotoxic doses. In in vivo studies, dendrimers decreased the PrP^Sc^ accumulation by more than 80% in mice spleen that occurred in the murine scrapie model [57]. As a follow-up to this experiment, the same dendrimer was also tested for its interaction with peptide prions. It has been found that the dendrimer inhibited the aggregation process of these peptides by slowing the formation of aggregates and reducing the number of amyloid fibrils. The study examined two peptides: PrP 185–208 was found to be structurally similar to the peptides found in Alzheimer’s disease, which has been linked to spongiform encephalopathies [57]. Fibril formation was directly inhibited by the interaction between the dendrimer and heparin.

MAP-Tau protein and β-amyloid peptides are the pathological aggregations to characterize Alzheimer’s disease. The 3rd and 4th generation cationic phosphorus dendrimers inhibited acetylcholinesterase activity, decreased TNF-a secretion and MAP-Tau aggregation processes, and had a weak β-amyloid antioxidant effects (Aβ1–28 peptide) [85]. An α-synuclein fibrillation decrease is a therapeutic approach for Parkinson’s disease by using the same dendrimers.

Neurodegenerative diseases were treated with a new compound, the interaction of viologen dendrimers with HSA (human serum albumin) has been examined, and it also had the inhibition of acetylcholinesterase and butyrylcholinesterase [86]. A viologen dendrimer was also shown to protect mHippoE-18 (embryonic mouse hippocampal cells) against insecticide, rotenone, nonselective piscicide, and pesticide. PAMAM and positive charge phosphorus dendrimers have also shown the same property [87].

#### 5.7.2. Anticancer agents

Anticancer properties are shown by multiple varieties of phosphorus dendrimers. The viologen dendrimers of 0 generations with PEG or ethylphosphonate were not toxic to normal cell line B14 fibroblasts and showed a toxic effect on N2a cancer cells. The dendrimers bearing phosphonate terminal groups at 20 mM showed that B14 cells had a viability of approximately 82% and N2a cells had a viability ofapproximately 37%. On the other hand, dendrimers bearing PEG terminal groups have approximately 91%, and approximately 41% of viability, respectively for B14 and N2a cells [70].

The structure of the previous compounds had shown unknown anticancer functions. In order to determine their anticancer properties, dendrimers containing many generations with different varieties of ethacrynic acid derivatives as terminal functions were synthesized. Additionally, ethacrynic acid possessed a low antiproliferative effect; despite inhibiting glutathione-S-transferase P1-1 was overexpressed in multiple cancer cells, and ethacrynic acid had less antiproliferative effect as well. Dendrimers of this type exhibited strong antiproliferative effects against liquid and solid tumors, demonstrating the dendritic effect efficacy. This 3rd generation dendrimer possessed an IC50 of 120 nM towards a solid KB cell line [88].

It is well known that platinum derivatives are effective anticancer drugs, especially cisplatin, which is the basis for metal complexes-based anticancer drugs. The phosphorus dendrimers surfaces were grafted with metallic derivatives that were predominantly used as catalysts, but some of them also exhibited anticancer properties [89]. In addition to catalyzing the allylic alcohol isomerization and alkynes hydration, the ruthenium complexes of PTA derivatives could also associate with supercoiled DNA to provide a relaxed DNA. According to the results of the initial screening, zero generations also had the same efficacy as cisplatin in test [90].

For copper complexing, dendrimers with pyridine imine derivatives were used as terminal groups, because N- and O-arylation and vinylation reactions afford efficient catalysts. Different types of dendritic complexes were tested against multiple human cancer cell lines, showing that 0.3 to 1.6 μM IC_50_ values have antiproliferative activity. N-(pyridin-2-ylmethylene)ethanamine complex copper functionalized G-3 dendrimer was most effective against cancer cells. In human cancer cell lines, cell death pathways were monitored in order to understand their mode of action: the detailed mechanism of action and the progression of apoptosis to secondary necrosis have been described [91].

A key component of innate immunity was human natural killer cells; it had major anticancer immunity, but the growth of cells was expensive and time-consuming. After three weeks in culture, 1st generation dendrimers with azabisphosphonate terminal functions could increase the number of natural killer cells by up to 500. In particular, carcinoma, leukemia cell lines, and natural killer cells were able to kill their target [92]. These natural killer cells were multiplied in the blood of both cancer patients and healthy volunteers with different myeloma because of the dendrimers [93].

#### 5.7.3. Against inflammation

Azabisphosphonate dendrimers will target the natural killer cells, but it was not the first target of dendrimers before it targeted the monocytes; it was also a key population of human innate immunity. In less than one minute, this dendrimer penetrated into monocytes and induced antiinflammatory responses [94]. By varying the numbers and types of the terminal group, a structure or activity relationship of the multiple properties of dendrimers has been developed. Dendrimers with azabisphosphonate terminal functions remained the most efficient dendrimers, but the structure of their internal cavities also had a critical role in biological applications. In an attempt to evaluate the dendrimer’s biological ability in rheumatoid arthritis, the antiinflammatory activation of this dendrimer was investigated. The inflammatory arthritis development was slowed down in mouse models, and antiosteoclastic activities were induced in mouse and human cells. The disease was suppressed by noncartilage destruction, and bone erosion, normal synovial membranes, and hind paw swelling decreased dramatically. The dendrimers were either given orally or injected with an analogous efficiency [95]. This dendrimer has also been tested in different inflammatory diseases, like neuroinflammation and uveitis, due to its very reassuring results as an antiinflammatory treatment for chronic diseases.

Serendipity discovered the dendrimers’ antiinflammatory properties. To cure acute inflammatory diseases, dendrimers were grafted with mannose derivatives and synthesized with the intention of finding new antiinflammatory drugs. Mycobacterium tuberculosis uses a mannose-capped lipoarabinomannan to reduce the host antiinflammatory responses, which resemble the supermolecular structure [96]. First to 3rd generation of dendrimers were synthesized with different mannose entities like 1, 2, or 3, which were attached to every terminal function, and monitored for their ability to bind DC-SIGN. The 3rd generation dendrimers capped with trimannosides showed the highest binding avidity. The compound significantly reduced acute lung inflammation in a mouse model when administered per se [97].

#### 5.7.4. Against ocular hypertension

The β-adrenoceptor antagonist is a nonselective carteolol that has limited agonist activity, and they are mostly used to reduce the patients elevated intraocular pressure with ocular hypertension or glaucoma [98]. By ionic interactions, a monomer complex with three carboxylic groups and phosphorus dendrimers carrying carboxylic groups at 6 or 12 terminal position has been synthesized to interact with carteolol [84,98]. Nanocarriers were used for carteolol ocular delivery, and rabbit models were used to treat in vitro for the three complexes as follows: 0, 1, and 2 generation molecules with Milli-Q water solution were administered into the rabbit eyes. After several hours of treatment using three compounds, no irritation was noticed in rabbit eyes. As compared with carteolol alone, the 2nd generation dendrimer showed 2.5 times higher penetration of complexed carteolol concentration inside the eyes [99].

#### 5.7.5. Against infections

In all members of *M. avium*, *M. tuberculosis*, and *M. bovis* a bacillus Calmette-Gue rin (BCG) complex and a mannose-capped lipoarabinomannan (ManLAM) would be present. During infection, ManLAM had a crucial role in the survival of mycobacterium. It played a role for both modulators, improving tuberculosis prevention and immunogen. In LPS-stimulated human dendritic cells (DCs), ManLAM slowed down the release of the proinflammatory cytokines by targeting the calcium-dependent C-type lectin receptor (CLR). Several viruses were recognized by DC-specific intercellular adhesion molecule 3-grabbing nonintegrin (DC-SIGN) [100].

Blattes et al. conducted an interesting study about the multiple types of phosphorus dendrimers’ development and design in order to mimic the bioactive supramolecular ManLAM structure, namely;

1. Mannose units like 12, 24, and 48 units were attached on the surface of G1, G2, and G3 varieties of phosphorus dendrimers;
2. Dimannose units were attached on the surface of G1-4 phosphorus dendrimers, corresponding to mannose units 12, 24, 48, and 96 per dendrimer; and
3. Trimannose units of G3 phosphorus dendrimers correspond to 48 units per dendrimer.

This demonstrated phosphorus dendrimers had the highest DC-SIGN binding affinity for 48 trimannoside caps, and 96 dimannosides were grafted into the 3rd generation phosphorus dendrimers; they also inhibited the proinflammatory TNFα cytokine production. In the acute lung inflammation mouse model, 3T phosphorus dendrimer containing 48 end trimannoside units was orally administrated (1 mg/kg). This reduced the neutrophil influx by DC-SIGN targeting, and in this experiment, the mouse model was exposed to aerosolized LPS [101].

## 6. Other applications

### 6.1. Biochips/Biosensors

In molecular medicine, the importance of microarray, like biosensors, is increasing. Generally, these devices have probes like nucleic acid immobilized on a glass slide (activated) at a discrete position. The fluorescence was detected by supramolecular interaction between the target and probes that are the complex mixtures of fluorescently labeled nucleic acids. The dendrimers improved stability and sensibility of detection by the 3-dimensional linkers between the probe and glass slide. In 1998 dendrimers were used commercially; in American hospitals, fluorimetric enzyme immunoassay analyzers were used first for quick detection of heart failure. Different types of dendrimers were used for biochips, like phosphorus dendrimers; these were currently available for a wide range of respiratory pathogens detection with high specificity and sensitivity. For the detection of Au nanoparticles and liposomes, the previous concept has been applied. Table 1 summarizes the phosphorus dendrimer-driven biosensing applications.

Different varieties of phosphorus dendrimers were used to develop biosensors in multiple ways. In order to fabricate nanotubes composed entirely of dendrimers, layer-by-layer (LbL) deposition of dendrimers with a positive charge (ammoniums) and negative charge (carboxylates) has been used, and various negatively charged quantum dots were added at certain layers, resulting in a graded band gap [102]. High-sensitivity detection of DNA hybridization was made by probe DNA, and the fluorescent Cy-5 was labeled to target DNA [103]. Selective detection and ultrahigh sensitivity of DNA hybridization by using negatively charged fluorescent core-shell polymers and ammonium phosphorus dendrimers in the LbL process was achieved [104]. In the LbL process, microcapsules were produced by the interaction between negative and positive charged dendrimers, and it was used for DNA hybridization detection [105].

Phosphorus dendrimers were particularly used in sensors to improve the sensitivity. Biosensors typically consist of two parts; the probe, which is an immobilized nucleic acid on surface-activated slides, and the nucleic acids labeled with fluorescent (target) is complex mixed with the sample. Hybridization (supermolecular) interaction between probes and targets is generally evaluated by fluorescence. In 1999 phosphorus dendrimers were used as biosensors with immobilization of HSA (human serum albumin) in the 5th generation dendrimer (3a-G5) have aldehyde terminal groups covalently linked to glass slides or amino-functionalized quartz [106].

A variety of generations of the dendrimer 3-G_n_ (CHO end groups and N_3_P_3_ core) were tested as spacers between the probe oligonucleotides and the solid surface. Generation 4 to 7 showed the best result for changing the signal-to-noise ratio. The 4th generation (3-G4) was selected because it was more easily synthesized than higher generations to enumerate probe/target supermolecular sensitivity. The Cy5-labelled 15 bases oligonucleotide, admiring the probe was hybridized in the concentrations (1 pM – 100 pM); 35-mer oligonucleotide was spotted in the glass slides with the dendrimer-functionalized slides and 12 functionalized glass slides were fabricated (commercially available). Fluorescent signals were quantified (only dendrislides) at the target concentrations of 0.001 nM of DNA. When compared to other functionalized glass slides, this dendrislide had 10 to 100 folds higher sensitivity detection [107].

### 6.2. Supports for cell cultures

The interaction on the cell surface was influenced by the morphology, charge, topography, and hydrophilicity, as reported in many studies. The glass surface modification would also modify the interaction with the cells. The generation 4 phosphorus dendrimers built by LbL process were obtained either by ammoniums or carboxylates terminal layer. By the positively charged dendrimers, the fetal rat cortical neurons attached better and matured faster on the surfaces [108]. Bifunctional Janus dendrimers were grafted on the surface of gold, showing that human osteoblast cells proliferate and adhere better to the negatively charged dendrimers. In positive-charged Janus, dendrimers were observed to elicit the cell apoptosis well [108].

### 6.3. Liposomal interaction

The interaction between the liposomes and phosphorus dendrimers was studied by Wrobel and colleagues in order to understand how dendrimers enter the cells. Cationic phosphorus dendrimers altered the thermotropy of bilayers by reducing phospholipid cooperativity. This effect strongly depended on the surface charge of the membrane and also influenced the lipid bilayers in both generations of dendrimers by decreasing the membrane fluidity. The dendrimers and cell membrane lipid bilayer interactions were analyzed by these data [109].

### 6.4. UTMD-based multivalent copper (II)-phosphorus dendrimers to kill cancer in vivo

Generation-3 phosphorus dendrimer attached with CuCl_2_ molecules (1G3-Cu) has been developed for noninvasive UTMD-promoted pancreatic chemotherapy and tumor MR imaging. The SW1990 pancreatic cancer cell growth was inhibited by 1.24 μM of IC_50,_ and it was sufficient for T1-weighted MR imaging with the r1 relaxivity of 0.7024 mM^–1^ s^–1^, shown by 1G3-Cu. Western blot and flow cytometry assay proved the 1G3-Cu chemotherapeutic effect. As a result, the 1G3-Cu was incubated with SW1990 cells for 24 h, and the apoptotic processes were activated through an increase in p53, phosphatase, Bax, and tensin homolog (PTEN) proteins, intracellular adenosine triphosphate (ATP) decrease, and Bcl-2 protein [113].

Through the induced sonoporation effect [108], penetration of drugs or NPs in a tumor was increased by UTMD technique. According to biodistribution studies, most of the 1G3-Cu uptake occurred in the liver, kidney, and lungs without UTMD; however, when UTMD was present, SW1990 tumors accumulated 1G3-Cu at a higher rate. On day 14, 1G3-Cu reduced tumor volume by 62%, compared to 35% without UTMD, demonstrating good anticancer effects. The hemolysis effect was not perceived with the range of 0.5 to 10 μM 1G3-Cu, and it showed superior activity without affecting the blood parameters, body weight, and major organs of mice.

### 6.5. Antimicrobial activities

Antibacterial and antifungal activity of these metallophosphorus dendrimers were tested against different strains like *S. aureus* (gram-positive bacteria), *E.coli*, *P.aeruginosa* (gram-negative bacteria), Candida albicans (yeast), and also drug resistance strains (*S. aureus* ZMF KSF, *Candida glabrata* ZMF SZP 4, *E. faecalis* ZMF BD 156). The compound’s minimal inhibitory concentration values were the low concentrations without measurable increases in optical density. There was no growth at the MBC values (minimal bactericidal concentrations) because the compound killed all cells. This indicated that the dendrimers had a broad fungicidal and bactericidal activity at the 3.5–500 mg/L concentration range [115].

### 6.6. Phosphorus dendrimers for multitarget

For antiapoptotic siRNAs (siBCL-2, siMCL-1, and siBCL-xL) carriers, researchers have designed and tested novel 3rd and 4th generation dendrimers with phosphorus-containing voluminous piperidine terminal cationic groups (AE2G3 and AE2G4). The dendrimers were examined in a multidrug approach involving the chemotherapeutic drug 5-FU (5-fluorouracil) to their potential to form siRNA complexes and deliver the genetic materials into the tumor cells, as well as it had the ability to inhibit cancer cell growth. HeLa cells were able to uptake 80–100% of the siRNA in the serum-containing medium through dendrimers of both generations compared to Lipofectamine, which was a widely used transfection agent, which showed only 40% uptake. AE2G3 dendrimers deliver siRNA cocktails at the 50 nM and 100 nM (low concentration) concentrations completely eliminating the cancer cells. AE2G3/siRNA cocktail complexes at low dose increased the 5-FU cytotoxic effect. It was demonstrated that the combined multitarget approach provided high potent serum-stable nanomaterials for gene-based drugs [116].

## 7. Combinational effect of phosphorus dendrimers

### 7.1. Gold and Phosphorus dendrimers

On the surface of the phosphorus dendrimer, Au (III) was substituted for Cu (II) in order to prepare new metallodendrimers. The antiproliferative activity increased 30-fold against HL-60 and KB by the conjugation of dendrimers with Au (II). The low antiproliferative activity of 1G3-[Au_48_] [AuCl_4_] (IC_50_ of 7.5 nM and 33 nM in the low nanomolar range on quiescent EPC cells was also notable as opposed to its strong antiproliferative activity against actively dividing cells. No further increase of cogency was observed in the antiproliferative activities against KB and HL-60 cells when Au (III) was applied to 1G3 at or above 10 Au (III). Furthermore, Cu (II) and Au (III) had no additional effects, suggesting there might be a threshold for Au-complexed dendrimers showing antiproliferative properties [117].

### 7.2. Viologen and phosphorus dendrimers

Viologen derivatives, also known as 4,4′-bipyridinium salts, were showing a large number of applications beyond their original application as herbicides [118]. Generally, this occurs because of their photoactive and electroactive properties and because they were able to form strong donor-acceptor complexes with electron-donating species. These substances affected the cell growth in an impressive way, especially in neuronal cells and immune blood cells like natural killer cells and monocytes, which played a key role in the fight against cancer and viral infections [119]. An in vivo study of phosphorus dendrimers showed their ability to deliver ocular drugs, the ability to visualize blood vessels in rat brains, and antiprion properties [120].

## 8. Toxicity

The cytotoxicity and cellular uptake of the dendrimers or surface-charged nanoparticles have been emphasized, and their toxicity is to be checked (Table 2). Higher cytotoxicity was observed generally in positively charged (ammonium groups have been attached on their surface) phosphorus dendrimers, and it also increased the drug delivery efficacy and cellular uptakes [121]. Different ammonium groups were attached to the surface, but the different types of phosphorus dendrimers with the ammonium groups were synthesized. Tertiary ammonium groups of phosphorus dendrimers with 3rd and 4rd generations did not cause a break in DNA that was isolated. Tertiary ammonium terminal groups induced lower cytotoxicity than the quaternary ammonium groups [122]. In the presence of dendrimers, the surface structure of human cells like HGFs, lymphocytes, and A549 cancer cell lines were changed together with nuclease condensation and membrane disruption [123]. The 2nd and 3rd generation phosphorus dendrimers of the same series showed high toxic effect in neuroblastoma cell line and hippocampal cells for 1 μM concentration, and it also showed disturbance in the activity of cells and ROS generation [124].

Phosphorus dendrimers were synthesized based on viologen units that preserve the positive charges in order to reduce cytotoxicity. The 0 generations of phosphonate group cytotoxicity were assessed against mouse hippocampal cells, where reduced ROS level and apoptosis at low level was observed [125]. Onion-peel dendrimers were synthesized by carbosilane and viologen units and ammonium groups at the end (primary). Cell viability of dendrimers was compared and measured on eukaryotic cells, and the outer surface charge (positive) showed toxicity [13].

The biological properties of 0 and 1st generation viologen-phosphorus dendrimers and 0 generations of dendrons were investigated, mainly concentrated on in vitro cytotoxicity, erythrocyte morphology, hemotoxicity, antibacterial activity, and membrane fluidity. Hemolysis occurred more frequently when dendrimers contained a high number of charges (viologen units). The 4th generation and 8th generation PEGylated dendrimers were not showing any toxicity to B14 cells, and against cancerous N2a cells it showed enhanced toxicity. The antimicrobial activity of the dendrimers was tested, and it showed good antibacterial activity towards *S. aureus* (gram-positive bacteria). Sixth and 7th generation dendrimers with a large number of positive charge and viologen units inhibited the growth of gram-negative bacteria (*E. coli* and *P. vulgaris*) [120].

The second way to reduce the toxicity was by preserving it in water; the solubility consisted of negatively charged grafting entities on the surface of the dendrimer. There was no toxicity observed with the terminal functions of azabisphosphonate and azabiscarboxylate towards human monocytes, while the terminal functions of vinyl carboxylate induced a high level of toxicity [75]. Another way to decrease toxicity was by using polyethyleneglycol terminal functions. At the surface of the dendrimers, PEG was linked with monophosphonate groups, and its toxicity was tested in human peripheral blood mononuclear cells. The phosphonate with PEG increased the viability of generation 1approximately 100%, and generation 3 was 95% compared with phosphonate without PEG [126].

## 9. Conclusion and future perspectives

A variety of phosphorus-containing dendrimers has been demonstrated to have the potential to fight against diseases, both at the macromolecular and cellular level, in this feature article. Phosphorus dendrimers were mostly carried out in in vitro experiments, but from that, only a few of the experiments were transferred into in vivo. Latter examples, 4th generation cationic dendrimers have been used in prion disease models, 1st generation anionic dendrimers have been used in rheumatoid arthritis and multiple sclerosis models, and mannose-capped 3rd generation dendrimers have been used in acute lung inflammation models. Mostly dendrimers without any special formulations were injected intravenously, but in two cases, they were given orally. Dramatic improvements in their health were observed in all cases when compared to untreated mice. It remains unclear whether these dendrimers have the ability to cross the BBB (blood-brain barrier) or not, despite these very positive results in vivo; further research into this was certainly required. In order to overcome “the large medical expectations for dendrimers, but less clinical translation are main features of the dendrimer paradox,” one of these dendrimers is undergoing preclinical requirements pertaining to “absorption, distribution, metabolism, excretion, and toxicity” (ADME-T) and “chemical manufacturing control” (CMC).

## Figures and Tables

**Table 1:** Applications of biosensors and copper (II)-phosphorus dendrimers.

SI. no.	Surface modification/dendrimers	Generation	Concentration	Bioreceptor	Detection	Applications	Ref.
1.	Biotin moieties	G1 and G4 biotin	0.5 to 1 mg mL^−1^	Streptavidin-modified gold nanoparticles	*E. coli* (foodborne pathogens)	Biotinylated phosphorus dendrimers were used as a control line in the NALF test.	[110]
2.	Gold substrates	-	30 pM and 50 pM on 1 bilayer and 4 bilayers	DNA probe	Limit detection of DNA hybridization	Immobilized probe DNA with Cy5 dye-labeled complementary target DNA was detected using SPFS.	[111]
3.	Amino-gold surface	G4	2.5 μM	DNA probe	Selective detection of protein-DNA interactions.	Interaction between SopB and SopC sequences could be discriminated by a dendrimer-grafted gold surface.	[107]
4.	Tailored surface	-	-	Amino-modified DNA probe	Fluorescent labelled oligonucleotides	High binding capacity for amino-modified oligonucleotides compared to commercially available aldehyde slides.	[112]
5.	Fluorescent phosphorus dendrimer	-	-	BM-derived macrophage (cytoplasmic tracer)	Transfected cells	It provides complementary and supportive insights into the retention and function of blood-borne phagocytes after spinal cord injury.	[80]
6.	Multivalent copper (II)-conjugated phosphorus dendrimers	1Gn-Cu, 2Gn-Cu, and 3Gn-Cu.	-	-	-	1G3 and 1G3-Cu demonstrated strong antiproliferative activities with IC50 values ranging from 0.3–1.6 μM.	[91]
7.	Copper (II) phosphorus dendrimers	1G_3_-Cu	-	-	-	It has an antiproliferative agent with a peculiar mode of action, through the Bax activation pathway, will induce apoptosis tumor cell death	[91]
8.	Poly (amidoamine) dendrimer-coordinated copper (II) complexes	Pyridine (Pyr)-functionalized G5.NHAc-Pyr/Cu (II)	4–10 μM	-	-	It is used to visualize tumors and their metastases through radiotherapy-enhanced T1-weighted MR imaging, as well as for radiochemotherapy treatment of both tumors and their metastases.	[50]
9.	Copper (II) metallodendrimers combined with pro-apoptotic siRNAs	G_1_- [CuCl_2_]_4_	3 μM	-	-	In breast cancer cells, apoptosis-inducing siRNAs targeting Mcl-1 and Bcl-2 effectively interact with Cu (II) dendrimers.	[113]
10.	Cu (II)-conjugated phosphorous-dendrimers	**G3B–**Cu (II)	-	-	-	It shows higher stability and antitumoral activities.	[114]

**Table 2:** Recent studies on the toxicity profile of the phosphorus dendrimers.

SI. No.	Dendrimers	Tested cells/animals	Studies	Toxicity	Reference
1.	Viologen phosphorus dendrimers (VPD1 and VPD2)	Mouse mHippoE-18 cells	Cell viability, ROS formation, cell death study, CAT activity, cell cycle analysis.	The toxicity of VPD1 is greater than that of VPD3. There was no significant cellular response to these two tested dendrimers, and only a low level of apoptosis was induced. VDPs indirectly reduce the level of ROS in cells.	[125]
2.	Water-soluble viologen (4,4′-bipyridyl) phosphorus dendrimers	Murine neuroblastoma cell line (N2a)	Cell viability, generation of ROS, mitochondrial activity, morphological modifications, and apoptosis and necrosis studies.	It shows only moderate cytotoxic, and some changes in mitochondrial function and ROS formation were detected. They will not promote necrosis and apoptosis. This shows that tested VPD is relatively safe for mouse neuroblast cells.	[127]
3.	Cationic Phosphorus dendrimers (pyrrolidinium amino group)	Mouse macrophage cell lines RAW264.7	Cell viability, cellular uptake	Pyrrolidinium surface groups demonstrated a stronger siRNA complexation, a having better biocompatibility, and higher cellular uptake.	[128]
4.	AzaBisPhosphorus dendrimer (ABP dendrimer)	Male cynomolgug monkeys *(M. fascicularis*)	Haematological, biochemical, clotting, and immunological parameters.	The ABP dendrimer was repeatedly injected into cynomolgus macaques and showed no adverse effects.	[129]
5.	3,4-diphenylmaleimide cationic phosphorus dendrimers	HeLa and A549 cells	Cell viability test	The cytotoxicity of this compound is relatively low towards HeLa and A549 cells. It indicates a relatively good tolerance of the cells after 24 h and a surprising cytotoxicity decrease after 48 h.	[79]
6.	Cationic phosphorus dendrimers	Murine embryonic hippocampal cells (mHippoE-18)	ROS, mitochondrial membrane potential alteration, morphology changes, apoptotic and cell death.	The two low generations (G2 and G3) are safe at a concentration of up to 1μM. Dendrimers modifying their surface with chemical groups will lower the cytotoxicity and help to improve their biomedical potential.	[130]
7.	Cationic phosphorus dendrimers	Mouse microglial cells and mouse neuroblastoma cells.	Cell viability test	The IC_50_ concentration of dendrimer causes a 50% decrease in cell viability; additionally, IC_10_ causes a 90% decrease, and IC_25_ causes a 75% decrease in the cell viability for BV-2 cells. The CPD G4 shows more cytotoxic to BV-2 cells than CPD G3.	[131]
8.	Cationic phosphorus dendrimers-Aβ_1-28_	N2a cells	Cell viability test.	During the aggregation process, samples were collected every 15 min and incubated with N2a cells. The high toxic effect was observed after 60 min, and then the toxicity was slowly reduced unless it reached a plateau value was reduced for the 135th minute.	[85]
9.	Polycationic phosphorus dendrimers	One healthy cell: HUVEC, and two cancerous cells: HEK 293 and HeLa	Cell viability test	2′-G4 and 3′-G4 dendrimers are almost noncytotoxic, whereas 4′-G4 dendrimers show high cytotoxic with healthy cells, and it has the propensity to increase the quantity of cancerous cells.	[132]
10.	Phosphorus dendrimer-based copper (II) complexes	SW1990 cells	Cell viability test	The cell viability decreases with increasing the Cu concentration of CuCl_2_ or 1G_3_-Cu complexes. The 1G_3_-Cu complexes display a higher antiproliferative effect than CuCl_2_. The higher number of cancer cells killed by the effect of 1G3-Cu complexes under UTMD.	[133]
